# Prevention practices for nonventilator hospital-acquired pneumonia: A survey of the Society for Healthcare Epidemiology of America (SHEA) Research Network (SRN)

**DOI:** 10.1017/ice.2021.427

**Published:** 2022-03

**Authors:** Dian L. Baker, Karen K. Giuliano

**Affiliations:** 1 School of Nursing California State University, Sacramento, California; 2 Center for Nursing and Engineering Innovation, Institute for Applied Life Sciences & College of Nursing, University of Massachusetts, Amherst, Massachusetts

Pneumonia is the leading hospital-acquired infection (HAI) among US hospitals, with nonventilator hospital-acquired pneumonia (NVHAP) now representing the majority of cases (65%).^1^ NVHAP affects ∼1 in 100 hospitalized patients across all risk factors and carries an associated crude mortality rate of 15%–30%.^2^ NVHAP is associated with increased antibiotic usage, high ICU utilization rates, and high readmission rates (20%) for survivors, and NHVAP is the most common pathway to sepsis.^2,3^ Despite the harm from NVHAP, with no current safety and reporting requirements, most hospitalized patients who acquire NVHAP do not receive therapeutic prevention interventions.^4^

In 2020, with the support of numerous healthcare leadership organization, the Department of Veterans’ Affairs formed the National Organization to Prevent Hospital-Acquired Pneumonia (NOHAP). The recently published NOHAP call to action outlines strategic areas for research focused on understanding the economic impact, pathogenesis, and best NVHAP survey methods. Also emphasized is the importance of describing pathways for prevention and implementation science related to NVHAP.^2^ The Association for Professionals in Infection Control and Epidemiology (APIC) published a published a position paper stating that hospitals should design local programs to address NVHAP, including surveillance and reporting requirements.^5^

To understand the current status of NVHAP prevention programs in US hospitals, we created a survey designed to identify current NVHAP policies and prevention practices.

## Methods

We conducted an Qualtrics survey of US members of the Society for Healthcare Epidemiology of America (SHEA)’s Research Network (SRN). The SRN is an international consortium of 96 hospitals that participate in multicenter healthcare epidemiology research. The 30-question survey was developed by the study authors in consultation with infection prevention experts and included questions on NVHAP prevention practices, policy, training, monitoring and documentation, family involvement, supply availability, and specific oral care practices related to the COVID-19 pandemic. An earlier version of the survey was field tested as part of a prior research study.^4^ We excluded pediatric network hospitals as NVHAP is less prevalent in children, resulting in 71 US qualified members. Data were collected between October 14, 2020, and April 2, 2021. Data were deidentified prior to forwarding the responses to the researchers. Data were exported from Excel (Microsoft, Redmond, WA) into SPSS version 26 software (IBM, Armonk, NY) for descriptive analyses. The study protocol was reviewed by the authors’ human subjects committee and determined to be exempt on the basis that the study related to quality improvement.

## Results

We e-mailed 71 members and received 33 completed surveys, for a 48% response rate: 28 (84.8%) were from academic centers, 4 (12.1%) were from community hospitals, and 1 was “other.” Respondents included both infection preventionists (n = 29, 87.9%) and epidemiologists (n = 4, 12.1%). Hospital bed size varied from 150 to 1,300 (Mean = 640) with geographic distributions of East (n = 19, 57.6%), Central (n = 8, 2.2%) and West (n = 6, 22%).

### NVHAP monitoring and prevention

Members indicated that they do not (1) monitor incidence of NVHAP (n = 8, 24.2%), (2) have a universal oral care policy (n = 19, 57.6%), (3) have a presurgical requirement for oral hygiene (n = 18, 54.5%), (4) routinely evaluate for continued need of oropharyngeal tubes (n = 22, 66.7%), and/or (5) track the antibiotic use associated with NVHAP (n = 24, 72.7 %,). Members reported that they do have (1) early mobility programs (n = 28, 84.8%), (2) programs to reduce use of stress ulcer prophylaxis (n = 24, 72%), and/or (3) routinely use the head of the bed in the up position (n = 28, 84.8%).

Most respondents reported a specific location in the electronic health record (EHR) for documentation of oral care frequency (n = 26, 78.8%), with 57.6% (n = 19) indicating that specific components of oral care are required in the documentation (eg, self care, dependent care, dentures). Most (n = 25, 75.8%) reported that there is a specific location in the EHR to record mobility type and frequency.

### Personnel, training, and oral care supplies

Just more than half of respondents reported that oral care competency training is required for staff (n = 19, 57.6%), and fewer than half of RNs had been trained to complete a swallow screen to determine whether more comprehensive assessment is required (n = 13, 39.4%). Furthermore, most hospitals did not have suction toothbrushes routinely available on medical-surgical units (n = 16, 48.5%) and 27% (n = 6) reported not providing basic oral care supplies. Regarding patient and family engagement in NVHAP prevention, only 21.2% (n = 7) support patient and family engagement in NVHAP prevention, and only 24.2% (n = 8) provide educational information about how to prevent NVHAP while in the hospital.

### NVHAP practices during the COVID-19 pandemic

Most SRN members do not track NVHAP as a secondary hospital-acquired infection in patients with COVID-19 (n = 25, 75.8%). Based on recent studies reporting effectiveness in eradicating SAR-CoV-2 in the oropharygrnal cavity,^6^ we asked about the use of antiseptic mouth rinse for patients and staff. Only 1 member reported the use of oral decontamination for patients and staff (n = 1, 3%).

## Discussion

The survey characterized current practices related to fundamental aspects of NVHAP prevention based on the NOHAP network’s call to action. The sample represented a consortium of geographically diverse and hospitals of various size. The members indicated that opportunities for quality improvement in NVHAP monitoring and prevention currently exist in US hospitals.

Based on these findings and the NOHAP Call to Action, hospitals may consider: (1) an in-depth review of current policies and practices related to NVHAP prevention, including patients with severe acute respiratory coronavirus virus 2 (SARS-CoV-2), (2) implementing robust staff training and professional development, (3) conducting a gap analysis to appraise type and quality of equipment and tools required to address NVHAP, (4) designing methods to capture NVHAP process measures and monitoring, and (5) providing patient and families with educational materials to help engage them in NVHAP prevention.

This study has several limitations. The survey was exploratory and designed to gather basic descriptive information regarding current US NVHAP prevention practices. The respondents were largely from academic hospitals and may not be representative of the NVHAP prevention practices of US healthcare facilities. Additionally, the survey was conducted during the COVID-19 pandemic, when variations in normal hospital practices and procedures likely occurred.

Hospitals are currently limited in their ability to track NVHAP due to a lack of clear guidelines and methodologies.^7^ Most hospitals are not tracking the incidence of NVHAP; however, most are prepared to monitor some key process measures (ie, oral care, mobility, tube care, etc) associated with NVHAP prevention. Individual hospital systems can determine NVHAP prevention quality measures and outcomes based recommendations from Association for Professionals in Infection Control and Epidemiology (APIC),^3^ Johns Hopkins’ I-COUGH,^8^ and Kaiser Permanente’s ROUTE bundle^9^ programs to address NVHAP. Notably, Kasier Permanente’s NVHAP prevention program was associated with a significant reduction in antibiotic use and highlighted the important role of mobility.^10^ Given the established harm from NVHAP, it is time for US hospitals to implement practice changes based on the evidence that does exist and to support additional research based on the NOHAP Call to Action.
